# Resting Cortisol Concentration as a Predictor of Illness Severity in Critically Ill Dogs With Systemic Inflammatory Response Syndrome

**DOI:** 10.1111/vec.70167

**Published:** 2026-09-25

**Authors:** Andreza Conti‐Patara, Linda G. Martin

**Affiliations:** ^1^ Department of Veterinary Clinical Sciences, College of Veterinary Medicine Washington State University Pullman Washington USA

**Keywords:** APPLE score, biomarker, critical illness

## Abstract

**Objective:**

To determine the use of resting serum cortisol concentration as a marker of outcome and illness severity as assessed by the Acute Patient Physiologic and Laboratory Evaluation (APPLE) full and fast scores in critically ill dogs with and without systemic inflammatory response syndrome (SIRS).

**Design:**

Prospective, observational study.

**Setting:**

University teaching hospital.

**Animals:**

Client‐owned dogs were enrolled into three different groups: SIRS (30 dogs), non‐SIRS (25), and healthy controls (19). Dogs with acute critical illness (<48 h) admitted to the ICU were enrolled and classified into SIRS and non‐SIRS groups. Healthy dogs were enrolled as controls. Resting serum cortisol concentrations were measured before treatment. APPLE full and fast scores were calculated at admission, with outcome recorded for each dog.

**Interventions:**

None.

**Measurements and Results:**

Resting serum cortisol concentrations were higher in the SIRS and non‐SIRS groups compared with the control group (*p* < 0.001); however, there was no significant difference (*p* = 0.59) between the SIRS and non‐SIRS groups. APPLE_full_ scores were higher in the SIRS group compared with the non‐SIRS (*p* < 0.0001) and control (*p* < 0.0001) groups. The APPLE_full_ scores increased by 1 (95% CI: 0.6–1.4) for every increase of 62.5 nmol/L (2.2 µg/dL) in resting serum cortisol concentration. Resting serum cortisol concentrations were not associated with mortality (*p* = 0.33). There was moderate evidence that APPLE_full_ scores can predict mortality (*p* = 0.03).

**Conclusions:**

Resting serum cortisol concentrations can be used as a marker of illness severity in critically ill patients with SIRS. Further studies enrolling larger numbers of cases may clarify its potential as a marker of outcome.

**Abbreviations**
APPLE: Acute Patient Physiologic and Laboratory Evaluation
CI: confidence interval
RI: reference interval
SIRS: systemic inflammatory response syndrome
SpO_2_
: pulse oximetry

## 1 Introduction

Resting serum cortisol concentration and its relationship to illness severity and mortality have been studied in general populations of critically ill people [1] and in people with specific critical conditions, such as trauma [2], acute brain injury [3], and sepsis [4], demonstrating a positive correlation between increased resting serum cortisol concentrations, increased mortality, and severity of illness. In dogs, the findings from the current literature are divergent. In dogs with babesiosis [5] and systemic inflammatory response syndrome (SIRS) [6], higher resting serum cortisol concentrations were associated with mortality, suggesting that resting cortisol concentrations may have prognostic use in certain disease states. Two prospective studies [7, 8] found increased resting plasma cortisol concentrations at the time of hospital admission in dogs with parvovirus enteritis compared with healthy dogs; however, there was no association between resting plasma cortisol concentration and patient mortality. A prospective study of 21 critically ill dogs found that admission plasma cortisol did not differ between survivors and nonsurvivors [9]. A recent retrospective study demonstrated a correlation between higher serum cortisol concentrations and nonsurvival [10]. There is currently a knowledge gap in veterinary medicine regarding the association between resting cortisol concentration, illness severity, and outcome in dogs with acute critical illness.

Identification of illness severity markers in critically ill patients aids in the recognition of patients at higher risk of mortality and allows more detailed investigation into pathologic processes. The qualities of the ideal biomarker include rapid kinetics (markers that rise quickly with an inducing stimulus), high sensitivity and specificity, simple identification by fully automated technology, short turnaround time, and availability as a point‐of‐care test with low production costs [11]. Resting serum cortisol concentration is an assay that fulfills most of these criteria, is inexpensive, and is available in many veterinary hospitals. However, the use of resting serum cortisol concentration as a predictor of the severity of illness and outcome in critically ill veterinary patients is still not fully known.

The purpose of the current study was to investigate the use of resting serum cortisol concentrations as a marker of illness severity and predictor of outcome in critically ill dogs with and without SIRS, as determined by the Acute Patient Physiologic and Laboratory Evaluation (APPLE) full and fast scores. This study aimed to investigate the relationship between resting cortisol concentrations and APPLE (full and fast) scores, evaluating the severity of the diseases in the three different groups (SIRS, non‐SIRS, and healthy dogs). Investigators hypothesized that critically ill dogs with SIRS would have the highest resting serum cortisol concentrations at the time of admission to the ICU, the highest APPLE full and fast scores, and the lowest survival rates compared with dogs without SIRS and healthy control dogs.

## 2 Materials and Methods

### 2.1 Animals

This was an observational, prospective study of dogs over 12 months of age with acute critical illnesses (illness present for <48 h) admitted to a university teaching hospital's small animal ICU from January of 2017 to October of 2018. The study was approved by the Institutional Animal Care and Use Committee, and all dogs were enrolled with written consent from their owners. Sixty client‐owned dogs were eligible for the study and were classified into SIRS and non‐SIRS groups based on the systemic inflammatory response variables of hypothermia (<38.1°C [100.6°F]) or hyperthermia (>39.2°C [102.6°F]), tachycardia (heart rate >120/min), tachypnea (respiratory rate >20/min), leukocytosis (>16 × 10^3^ cells/µL), leukopenia (<6 × 10^3^ cells/µL), or >3% bands on the WBC count. Based on the definition, patients with two or more criteria and an appropriate underlying condition are considered to have SIRS [12, 13]. To enhance the identification of critically ill patients, the current study considered patients with three or more SIRS variables to have SIRS [14]. Dogs that received exogenous corticosteroids, etomidate, or ketoconazole within the last 4 weeks, and dogs with known malignancies or diseases that involved the pituitary or adrenal glands, were excluded from the study in accordance with the exclusion criteria of a previous study [15]. Five dogs were euthanized shortly after ICU admission due to financial constraints and were excluded from the study, leaving 30 dogs in the SIRS group and 25 dogs in the non‐SIRS group. Additionally, 19 healthy client‐owned dogs that presented to the university teaching hospital's Community Practice service were enrolled as controls. These dogs were presented for elective periodontal treatment, and the relevant data were obtained on the preanesthetic evaluation. The same eligibility criteria were used for the control group as the SIRS and non‐SIRS groups. Dogs were classified as healthy based on history, physical examination, CBC, and serum biochemistry results. A power analysis was conducted before the study, indicating that 150 dogs should be included and distributed equally across the groups. However, due to an insufficient caseload and a specific inclusion criterion, a total of 74 dogs were ultimately enrolled.

### 2.2 Data Collection

Signalment, previous medical history, and physical examination findings at hospital admission were recorded. Blood samples were collected at the time of enrollment and before any treatment for CBC^1^, serum biochemistry panel^2^, and blood glucose, blood lactate^3^, and resting serum cortisol concentrations^4^. Pulse oximetry^5^ (SpO_2_) value, mentation score, and ultrasound^6^ body cavity fluid scores were also obtained at admission. The APPLE full and fast scores were calculated according to a previous report [16]. The APPLE_full_ was calculated using serum creatinine concentration, WBC count, serum albumin concentration, SpO_2_, serum total bilirubin concentration, mentation score, respiratory rate, age, body cavity fluid score, and blood lactate concentration. The APPLE_fast_ score was calculated using blood glucose, serum albumin, and blood lactate concentrations, platelet count, and mentation score. All scores were calculated using data obtained upon presentation to the hospital.

### 2.3 Sample Handling

Six milliliters of blood were drawn from each dog via peripheral venipuncture. Two milliliters were placed in an EDTA tube for the CBC and 4 mL in a plain tube with no additives for the biochemistry profile and cortisol assay. After a firm clot had formed, the plain tube was centrifuged at 197 × *g* for 10 min to separate the serum from the RBCs. For the cortisol assay, 1 mL of serum was decanted from the spun sample and frozen at −80°C (−112°F) until analysis.

### 2.4 Resting Serum Cortisol Concentration Assay

Resting serum cortisol concentrations were measured using a commercially available solid‐phase, competitive chemiluminescent enzyme immunoassay that has been previously validated [17]. The lower limit of analytical sensitivity was 14 nmol/L (0.5 µg/dL). Reported interassay and intraassay coefficients of variation for the chemiluminescent enzyme immunoassay were >20% at 0.25 µg/dL (6.9 nmol/L) but ≤20% at values ranging from 14 to 690 nmol/L (0.5–25 µg/dL) [17]. The samples from the different groups (SIRS, non‐SIRS, and healthy groups) were randomly mixed and analyzed in multiple batches. All samples were assayed in duplicate. The normal reference interval (RI) for resting serum cortisol concentration by this assay was 20–160 nmol/L (0.7–5.8 µg/dL).

### 2.5 Outcome

Outcome was recorded as survival to hospital discharge or nonsurvival. All dogs that did not survive died of natural causes; dogs that were euthanized were excluded from the study. The treatment of each dog enrolled in the study was determined by its primary clinician.

### 2.6 Statistical Analysis

Statistical analyses were performed using freely available statistical software^7^. Resting serum cortisol concentrations, APPLE_full_, and APPLE_fast_ scores were evaluated for normal distribution using the Shapiro–Wilk test. Because the variables were not normally distributed, the Kruskal–Wallis test, a nonparametric test, was used to determine the difference between resting serum cortisol concentrations among the different groups (SIRS, non‐SIRS, and healthy controls). A post hoc comparison was performed using the Mann–Whitney *U*‐test to conduct a pairwise comparison of resting serum cortisol concentrations among different groups. A linear regression model was fit to relate APPLE_full_ and APPLE_fast_ scores to the group (SIRS, non‐SIRS, and healthy controls) and resting serum cortisol concentrations. To account for a nonlinear relationship between resting serum cortisol concentrations and the APPLE_full_ and APPLE_fast_ scores, a natural cubic spline with three knots was used for resting serum cortisol concentrations and was interacted with the group factor. The model with three knots was selected based on the visual inspection against observed data and residual plots to inspect model fitness. An *F*‐test was conducted on the fitted model to evaluate the effect of the group and a basal serum cortisol concentration on APPLE_full_ and APPLE_fast_ scores. This was followed by a post hoc Tukey test to estimate pairwise comparisons among groups. The likelihood ratio drop test was applied to evaluate the association between resting serum cortisol concentrations and APPLE_full_ and APPLE_fast_ scores with the odds of mortality. For all statistical analyses, *p* ≤ 0.05 was considered significant.

## 3 Results

This prospective observational study enrolled 74 client‐owned dogs into three different groups: SIRS (30 dogs), non‐SIRS (25 dogs), and healthy controls (19 dogs). The median ages of dogs in the SIRS, non‐SIRS, and control groups were 7.6 years (1–13.3 years), 7.2 years (1–13 years), and 7.8 years (2–12.4 years), respectively. There was no significant difference in the age of the dogs in each of the groups.

The three groups were made up of a diverse collection of breeds. The SIRS group comprised three each of the following breeds: mixed breed dogs, Australian Cattle Dogs, and Pugs. Additionally, there were two Golden Retrievers and one each of the following breeds: Weimaraner, German Shepherd Dog, Irish Setter, Great Dane, Bloodhound, Cardigan Welsh Corgi, Standard Poodle, Labrador Retriever, Miniature Pinscher, Basset Hound, Redbone Coonhound, Pomeranian, Alaskan Malamute, Siberian Husky, Border Collie, Havanese, English Setter, and Scottish Terrier. Thirteen dogs were neutered females, four were intact females, eight were neutered males, and five were intact males. The non‐SIRS group comprised three mixed breed dogs, two each of Miniature Poodles and Golden Retrievers, and one each of the following breeds: Chesapeake Bay Retriever, Russian Wolfhound, Boxer, Brussels Griffon, Australian Cattle Dog, Collie, Miniature Schnauzer, Labrador Retriever, Great Dane, Airedale Terrier, Brittany Spaniel, Chihuahua, Giant Schnauzer, American Pit Bull Terrier, Doberman Pinscher, Leonberger, Scottish Terrier, and Rhodesian Ridgeback. The non‐SIRS group had 12 neutered females, two intact females, seven neutered males, and four intact males. The control group included two each of the following breeds: mixed breed dogs, Jack Russell Terriers, and Schnauzers, and one each of the following breeds: Pomeranian, Labrador Retriever, Chihuahua, Siberian Husky, Shih Tzu, German Shorthaired Pointer, American Staffordshire Terrier, French Bulldog, Yorkshire Terrier, Golden Retriever, American Pit Bull Terrier, Fox Terrier, and Leonberger. In this group, there were nine neutered females, one intact female, eight neutered males, and one intact male.

Within the SIRS group, five dogs were diagnosed with trauma (two with blunt trauma and three with head trauma), four with pyothorax, three with respiratory distress (one dog each with pulmonary histiocytic sarcoma, acute respiratory distress syndrome, and noncardiogenic pulmonary edema due to upper airway obstruction), four with septic peritonitis, two with pyometra, two with gastric dilatation–volvulus, and one dog of each with hemoabdomen, septic shock, pancreatitis, necrotizing fasciitis, cluster seizures, gastrointestinal foreign body, diabetic ketoacidosis, bile peritonitis, hemorrhagic diarrhea, and cholangiohepatitis. Within the non‐SIRS group, seven dogs were diagnosed with trauma (one dog each with pelvic fractures, spinal cord subluxation between T13 and L1, right carpus fracture and degloving injury, penetrating chest wound injury and rib fracture, cervical soft tissue laceration, pneumothorax and lung contusion due to blunt trauma, and pneumothorax and rib fractures), three with gastric dilatation–volvulus, two with spontaneous pneumothorax, and one dog each with pneumonia, acute respiratory distress syndrome, severe pulmonary hypertension, left‐sided congestive heart failure, liver dysfunction, cluster seizures due to a brain tumor, partial colonic torsion, heat stroke, hemorrhagic diarrhea, acute kidney injury due to leptospirosis, gastrointestinal foreign body, abscess on the right front limb, and pericardial effusion. Within the control group, all dogs were healthy and admitted for pre‐evaluation for periodontal treatment.

Twenty‐four of 30 dogs in the SIRS group and 22 of 25 dogs in the non‐SIRS group had resting serum cortisol concentrations above the normal RI (20–160 nmol/L). Only one of 19 dogs in the healthy control group had a resting serum cortisol concentration above the RI. No dogs involved in the study had a resting serum cortisol below the RI.

A significant impact of the group (*p* < 0.001) on resting serum cortisol concentrations was observed on the Kruskal–Wallis test. At admission, resting serum cortisol concentrations were higher in the SIRS group (352 nmol/L; range: 73–1380 nmol/L [12.7 µg/dL; range: 2.6–50 µg/dL]) compared with the non‐SIRS (208.5 nmol/L; range: 74–861 nmol/L [7.5 µg/dL; range: 2.6–31.2 µg/dL]) and control (70 nmol/L; range: 42–242 nmol/L [2.5 µg/dL; range: 1.5–8.7 µg/dL]) groups. Post hoc analysis using Mann–Whitney *U*‐test revealed that the resting serum cortisol concentrations were higher in the SIRS and non‐SIRS groups compared with the control group (*p* < 0.001 and *p* < 0.001, respectively); however, there was no difference between the SIRS and non‐SIRS groups (*p* = 0.59) (Figure 1).

**FIGURE 1:**
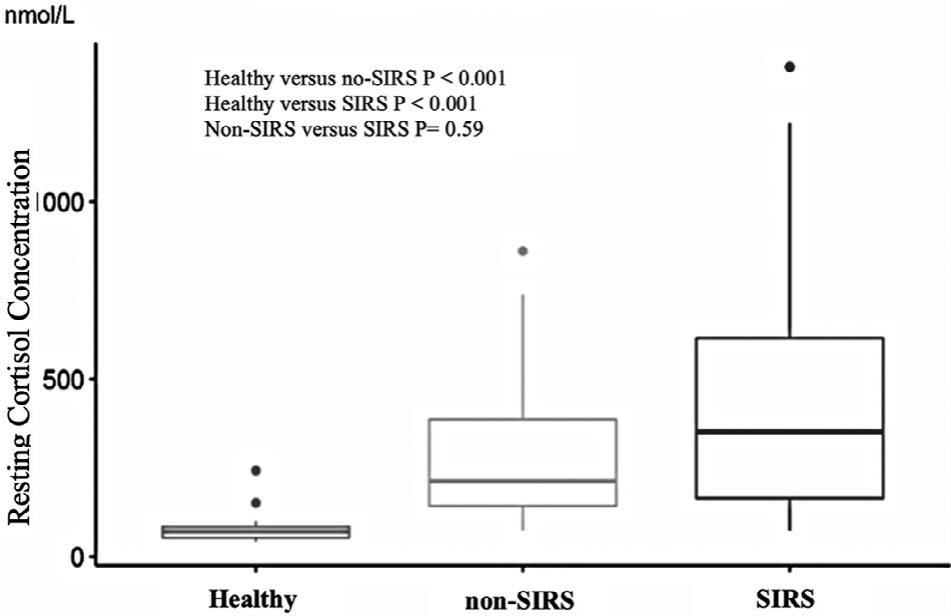
Median resting cortisol concentrations (mmol/L) in 19 healthy dogs, 25 dogs with acute critical illness but no SIRS, and 30 dogs with acute critical illness and SIRS. Resting cortisol concentration was higher in the SIRS and non‐SIRS groups compared with the healthy group (*p* < 0.001 and *p* < 0.001, respectively). Resting serum cortisol concentration was not different between the SIRS and the non‐SIRS groups (*p* = 0.59). SIRS, systemic inflammatory respiratory syndrome.

Twelve of 30 dogs in the SIRS group and four of 25 dogs in the non‐SIRS group died. Resting serum cortisol concentrations tended to be higher in nonsurvivors (452.50 nmol/L; range: 162–1380 nmol/L [16.4 µg/dL; range: 5.8–50 µg/dL]) compared with survivors in the SIRS group (274.5 nmol/L; range: 73–1222 nmol/L [9.9 µg/dL; range: 2.6–44.2 µg/dL]); however, they were not significantly different (*p* = 0.1). In the non‐SIRS group, resting serum cortisol concentrations were the same in nonsurvivors (208.5 nmol/L; range: 121–494 [7.5 µg/dL, range: 4.3–17.9 µg/dL]) as in survivors (219 nmol/L; range: 74–861 [7.9 µg/dL; range: 2.6–31.2 µg/dL]; *p* = 0.9). The likelihood ratio tests were performed to assess the significance of the predictive outcomes of APPLE_full_ and APPLE_fast_ scores and resting serum cortisol concentration and to compare them with the model with only the group. There was no evidence that including APPLE_fast_ (*p* = 0.14) or resting serum cortisol concentration (*p* = 0.33) improves outcome prediction compared with a model with group factor alone. There was moderate evidence that including APPLE_full_ scores (*p* = 0.03) improves predictions of mortality. A linear regression model was fit to relate the APPLE_full_ score to the three groups and resting serum cortisol concentration at the time of hospital admission (Table 1). The *F*‐tests provide strong evidence of main effects of cortisol (*p* = 0.0003) and group effects (*p* = 0.0002) but no significant effect of an interaction between resting cortisol and groups and no effect of a nonlinear resting serum cortisol on APPLE_full_ scores (*p* = 0.2278).

**TABLE 1:** Linear regression analysis of APPLE_full_ scores in dogs across SIRS (30 dogs), non‐SIRS (25 dogs), and healthy control (19 dogs) groups, with resting cortisol concentration modeled using restricted cubic splines, including their interaction effects.

Parameter	Estimate	SE	*t* value	Pr (>|*t*|)
Intercept	17.9129	6.0661	2.95	0.0044
Resting cortisol [1]	−0.0439	0.0854	−0.51	0.6085
Resting cortisol [2]	1.996	1.9712	1.01	0.315
Non‐SIRS group	0.3839	7.6377	0.05	0.9601
SIRS group	8.7163	7.1791	1.21	0.2291
Resting cortisol [1] × Non‐SIRS group	0.0938	0.0888	1.06	0.2947
Resting cortisol [2] × Non‐SIRS group	−2.1776	1.9737	−1.1	0.2739
Resting cortisol [1] × SIRS group	0.0667	0.0871	0.77	0.4469
Resting cortisol [2] × SIRS group	−2.0087	1.9719	−1.02	0.3121

*Note*: The model parameter estimates, SE, *t* value, and *p* values from the linear regression of APPLE_full_ scores across the groups, resting cortisol (using restricted cubic spline), and their interaction. Adjusted *R*‐square = 0.59. The restricted cubic spline term for resting cortisol used three knots, resulting in 2 degrees of freedom.

Abbreviations: APPLE, Acute Patient Physiologic and Laboratory Evaluation; SIRS, systemic inflammatory respiratory syndrome.

There was a difference in the APPLE_full_ scores among the three groups (*p* < 0.001). The APPLE_full_ scores were higher in the SIRS group (35.8; range: 15–61) compared with the non‐SIRS (27.4; range: 17–47; *p* < 0.0001) and control (15.5; range: 7–27; *p* < 0.0001) groups. The APPLE_full_ scores increased by 1 (95% confidence interval [CI]: 0.6–1.4) for every increase of 62.5 nmol/L (2.2 µg/dL) in resting serum cortisol concentration (Figure 2).

**FIGURE 2:**
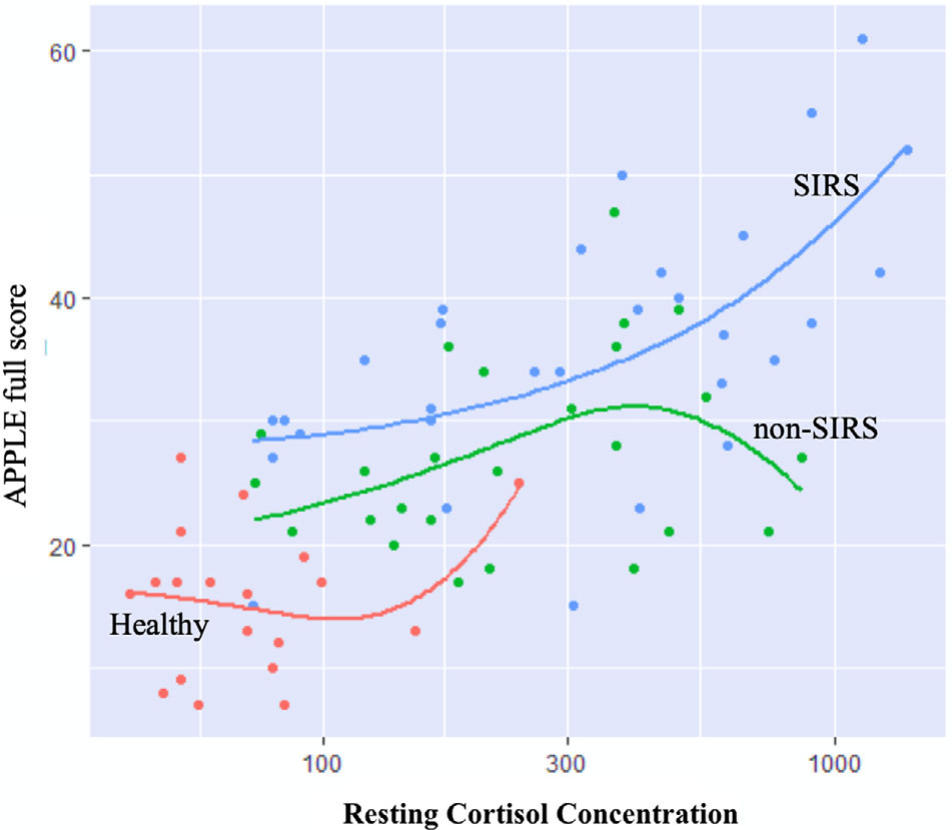
Linear regression of resting serum cortisol concentration and APPLE_full_ score in 19 healthy dogs, 25 dogs with acute critical illness but no SIRS, and 30 dogs with acute critical illness and SIRS. In the SIRS group, the APPLE_full_ score increases by an average of 4.89 (95% CI: 2.89–6.89) for every increase in cortisol of 1 SD. APPLE, Acute Patient Physiologic and Laboratory Evaluation; CI, confidence interval; SIRS, systemic inflammatory respiratory syndrome.

A linear regression model was also fit to relate the APPLE_fast_ score to the three groups and resting serum cortisol concentration at the time of hospital admission (Table 2). There was a difference in APPLE_fast_ scores among the three groups (*p* = 0.0002). The APPLE_fast_ scores for SIRS, non‐SIRS, and control groups were 25.1 (range: 10–34), 20.6 (range: 12–32), and 12.8 (range: 7–20), respectively. A post hoc Tukey test showed APPLE_fast_ scores were higher in the SIRS group compared with both the non‐SIRS (*p* = 0.003) and control (*p* < 0.0001) groups. The APPLE_fast_ scores were also higher in the non‐SIRS group compared with the control group (*p* < 0.0001). APPLE_fast_ scores increased by 0.1 (95% CI: −0.2 to 0.4) for every increase of 62.5 nmol/L (2.2 µg/dL) in resting serum cortisol concentration (Figure 3).

**TABLE 2:** Linear regression of APPLE_fast_ scores in dogs across SIRS (30 dogs), non‐SIRS (25 dogs), and healthy controls (19 dogs), with resting cortisol concentration modeled using restricted cubic splines, including their interaction effects.

Parameter	Estimate	SE	*t* value	Pr (>|*t*|)
Intercept	16.1057	4.3541	3.7	0.0004
Cortisol [1]	−0.0484	0.0613	−0.79	0.4329
Cortisol [2]	0.9454	1.4148	0.67	0.5064
Non‐SIRS group	0.1007	5.4821	0.02	0.9854
SIRS group	6.47	5.1529	1.26	0.2138
Cortisol [1] × Non‐SIRS group	0.0723	0.0638	1.13	0.261
Cortisol [2] × Non‐SIRS group	−1.0311	1.4166	−0.73	0.4693
Cortisol [1] × SIRS group	0.0603	0.0625	0.96	0.3386
Cortisol [2] × SIRS group	−0.9794	1.4154	−0.69	0.4914

*Note*: The model parameter estimates, SE, *t* value, and *p* values from the linear regression of APPLE_fast_ scores across the groups, resting cortisol (using restricted cubic spline), and their interaction. Adjusted *R*‐square = 0.44. The restricted cubic spline term for cortisol used three knots, resulting in 2 degrees of freedom.

Abbreviations: APPLE, Acute Patient Physiologic and Laboratory Evaluation; SIRS, systemic inflammatory respiratory syndrome.

**FIGURE 3:**
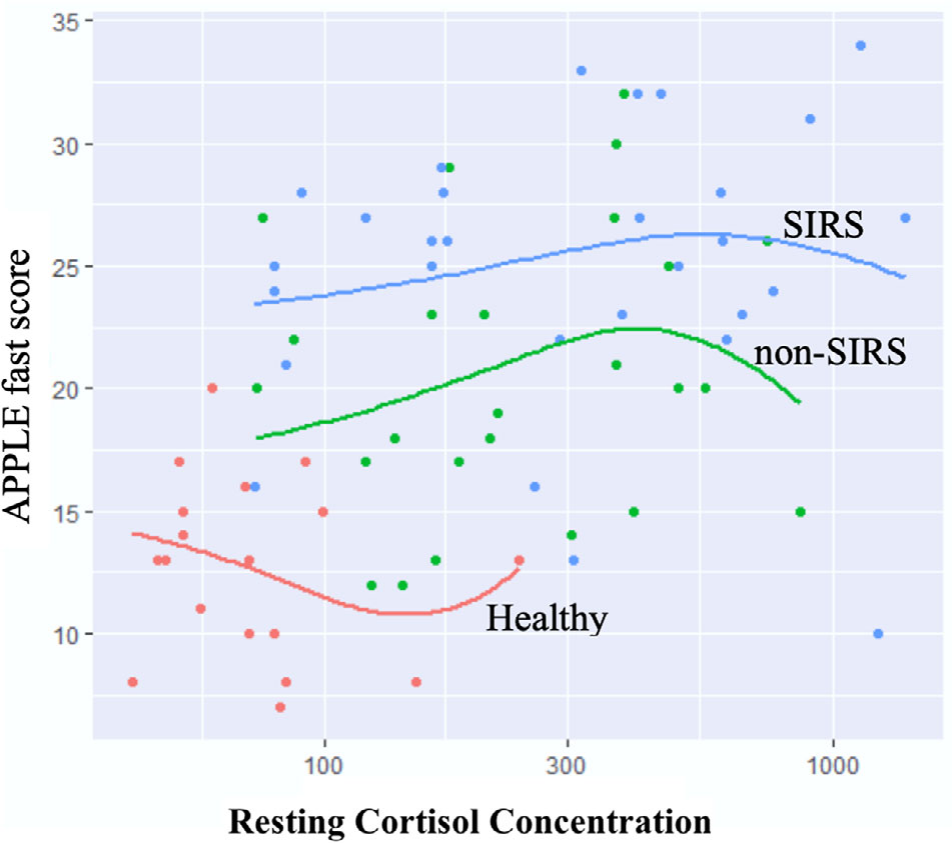
Linear regression of resting serum cortisol concentration and APPLE_fast_ score in 19 healthy dogs, 25 dogs with acute critical illness but no SIRS, and 30 dogs with acute critical illness and SIRS. The APPLE_fast_ score increased by an average of 0.50 (95% CI: −0.96 to 1.94) for every increase in cortisol of 1 SD. APPLE, Acute Patient Physiologic and Laboratory Evaluation; CI, confidence interval; SIRS, systemic inflammatory respiratory syndrome.

In the SIRS group, the APPLE_full_ scores were higher in nonsurvivors (39.5; range: 23–55) compared with survivors (32.5; range: 15–61; *p* = 0.02). In the non‐SIRS group, APPLE_full_ scores were not different in nonsurvivors (30; range: 18–39) compared with survivors (26; range: 17–38; *p* = 0.6).

The APPLE_fast_ scores were not different (*p* = 0.06) when comparing nonsurvivors (27.5; range: 23–33) and survivors (24.5; range: 10–34) in the SIRS group. In the non‐SIRS group, the APPLE_fast_ scores were also not different (*p* = 0.7) when comparing nonsurvivors (19; range: 17–23) and survivors (20; range: 12–32).

## 4 Discussion

This current study demonstrated increased resting serum cortisol concentrations in critically ill dogs with SIRS and those with non‐SIRS disease states compared with healthy control dogs. Twenty‐four of 30 dogs with SIRS and 22 of 25 dogs with non‐SIRS diseases had resting serum cortisol concentrations above 160 nmol/L (RI: 20–160 nmol/L; 5.8 µg/dL [RI: 0.7–5.8 µg/dL]). Several studies in dogs [5, 6, 10, 15, 18] and in people [2, 4, 19] have reported similar findings. The increased resting serum cortisol concentrations in critically ill populations compared with controls is in part due to the intense adrenocortical response to stress and inflammation. Alteration in cortisol metabolism may contribute to this finding as well. In people, the enzyme 11β‐hydroxysteroid dehydrogenase type 2, which converts cortisol to cortisone, was found to be decreased in critically ill populations compared with controls, leading to hypercortisolemia in critically ill patients [20]. A recent prospective study assessed cortisol metabolism in critically ill dogs with sepsis compared with healthy dogs. The findings revealed that critically ill dogs exhibit reduced activity of 1β‐hydroxysteroid dehydrogenase type 2, resulting in decreased cortisol metabolism. This observation is consistent with previously documented findings in people. Although the current study did not explore the underlying causes of hypercortisolism, its results reinforce the existing literature regarding hypercortisolemia in critical illness.

Predictors of outcome may be useful in identifying critically ill dogs likely to survive their illness. This information can be helpful to doctors when it comes to deciding a course of therapy and providing owners an accurate prognosis for their pets [21]. Assessment of resting cortisol concentration is inexpensive and accessible in most practices, making it a valuable tool for comprehensive patient assessment. In people, studies involving patients with community‐acquired pneumonia [19], trauma [2], craniocerebral injury [3], and critically ill children [4] found that patients with increased resting serum cortisol concentrations on presentation to the ICU had higher mortality compared with patients with resting cortisol concentrations within the normal range. In agreement with the studies in people [2, 3, 4, 19], a study involving dogs with babesiosis found that dogs with increased resting serum cortisol concentrations at hospital admission also had higher mortality rates [5]. In another study of dogs with SIRS, resting serum cortisol concentrations (>138 nmol/L; >5 µg/dL) were associated with higher mortality [6]. In a retrospective study involving 229 critically ill dogs, increased resting serum cortisol concentration was associated with nonsurvival to hospital discharge [10]. In contrast with these studies, two studies [7, 8] evaluating dogs with parvoviral enteritis found no statistical difference between serum cortisol concentration at admission to the ICU in nonsurvivors compared with survivors. A similar finding was observed in a study involving a population of dogs with sepsis and septic shock [18].

The current study also showed that nonsurvivors often presented with numerically higher resting serum cortisol concentrations than survivors. However, no statistical difference was found between the two groups, which agrees with previous studies [7, 8, 18]. Resting serum cortisol concentrations did not predict mortality, likely due to the variability of disease processes and the small number of cases enrolled in each group. Serum cortisol concentration was a good predictor of outcome in dogs with parvoviral enteritis when resting serum cortisol was measured at 24 and 48 h of hospitalization [8] and at 72 h of hospitalization [7]. The current study did not evaluate resting serum cortisol concentrations at different time points; however, it may indicate that resting serum cortisol concentrations measured at times other than admission to the hospital may be more helpful in determining patient outcomes.

The APPLE_full_ and APPLE_fast_ scores were developed to evaluate the level of illness in a population of hospitalized dogs, reflecting the severity of derangement of normal physiology identified by abnormalities in clinical and laboratory variables and correlating it with the likelihood of survival to hospital discharge. These scoring systems are often used to enroll patients in clinical research but are also helpful in developing protocols in triage and therapy [16]. In the current study, the APPLE_full_ and APPLE_fast_ scores were higher in the SIRS group than in the non‐SIRS group, indicating that patients in the SIRS group, including those with three or more SIRS variables, had higher illness severity scores. The SIRS criteria have been thoroughly assessed in earlier studies and have shown limited effectiveness in differentiating between healthy and diseased dogs, exhibiting a weak correlation with clinical outcomes [22, 23]. A study including 179 human patients evaluated the number of SIRS criteria present at hospital admission found that patients with three or more SIRS criteria were more likely to require intensive care and had worse outcomes [14]. Possibly the inclusion of at least three SIRS variables enhances the identification of critically ill dogs, as previously observed in people [14]. However, making such a distinction was not the aim of the current study, and future studies evaluating the sensitivity and specificity of the SIRS criteria, including three or more variables to distinguish critically ill dogs, are necessary to confirm this finding.

Considering the APPLE scores and the resting serum cortisol concentrations, increased resting serum cortisol concentrations were positively related to the APPLE_full_ score, demonstrating the association between higher resting cortisol concentrations and severity of disease. The APPLE_full_ score increased by 1 for every increase of 62.5 nmol/L (2.2 µg/dL) in resting serum cortisol concentration at the time of hospital admission. The APPLE_fast_ score was also positively related with resting serum cortisol concentrations but with a smaller numerical impact. The APPLE_fast_ score increased by 0.1 for every 62.5 nmol/L (2.2 µg/dL) in resting serum cortisol concentration. Similar results were seen in two different studies involving dogs in shock [24] and those with gastric dilatation–volvulus [25], where the APPLE_fast_ score was not predictive of survivors versus nonsurvivors. In the current study, the association between resting serum cortisol concentrations and illness severity was assessed using the APPLE_full_ score, suggesting that resting cortisol may have an application in determining the severity of acute illness in critically ill dogs.

Comparing the two APPLE scores between survivors and nonsurvivors in the current study, both scores were higher in nonsurvivors. However, only the APPLE_full_ scores were significantly higher in the nonsurvivors group. The APPLE_full_ score has been reported as an accurate predictor of prognosis in different populations of critically ill dogs, such as patients with myocardial injury [26] and patients undergoing mechanical ventilation, in which lower scores were associated with better outcomes [27].

Resting serum cortisol concentrations were not significantly different when comparing dogs with SIRS and non‐SIRS diseases. Similar to our study, serum cortisol concentrations were also not significantly different between dogs with sepsis and septic shock in a recent study [18]. However, the smaller sample size in the current study may have led to a type II error, potentially contributing to discrepancies in the results compared with previous studies. These dogs had higher cortisol concentrations, but this was not statistically significant because of the large interindividual variability in critically ill patients, inherent heterogenicity, and smaller sample size. The limited sample size may have reduced the statistical power to detect meaningful differences, representing a limitation of this study.

In conclusion, the findings of the current study demonstrate the importance of measuring resting serum cortisol concentration in critically ill dogs. Patients with higher APPLE_full_ scores also had higher resting serum cortisol concentrations, suggesting that its assessment may help identify critically ill dogs at an increased risk of mortality. Further studies in large populations with a higher proportion of nonsurvivors may increase statistical power and clarify its potential as a marker of outcome and survival. Additionally, this study highlights the prevalence of hypercortisolism in critically ill dogs. Both hypercortisolism and critical illness‐related corticosteroid insufficiency have been reported in this population, emphasizing the necessity for larger multicenter studies to explore their interrelationship and clinical significance in critically ill patients.

## Author Contributions

**Andreza Conti‐Patara**: investigation, writing – original draft, methodology, visualization, writing – review and editing, data curation, formal analysis, conceptualization. **Linda G. Martin**: conceptualization, funding acquisition, writing – original draft, methodology, visualization, writing – review and editing, investigation, formal analysis, project administration, supervision, data curation, resources.

## Conflicts of Interest

The authors declare no conflicts of interest.

## Endnotes

1 ADVIA120 Hematology, Siemens Healthcare GmbH, Erlangen, Germany.

2 Dimension Xpand Plus chemistry analyzer, Siemens Healthcare GmbH.

3 Lactate Plus, Nova Biomedical, MA, USA.

4 Cortisol Assay Immulite 1000, Siemens HealthCare Diagnostics Products, Los Angeles, CA.

5 Pulse oximetry, Masimo Rad 57, Masimo, Irvine, CA.

6 Point‐of‐care ultrasound, Sonosite SII, Fujifilm Sonosite, Bothell, WA.

7 R (version 4.4.2). R Core Team (2018). R: A language and environment for statistical computing. R Foundation for Statistical Computing, Vienna, Austria. https://www.R‐project.org/.
